# Network Homophily and the Evolution of the Pay-It-Forward Reciprocity

**DOI:** 10.1371/journal.pone.0029188

**Published:** 2011-12-15

**Authors:** Yen-Sheng Chiang, Nobuyuki Takahashi

**Affiliations:** 1 Department of Sociology and Institute for Mathematical Behavioral Science, University of California Irvine, Irvine, California, United States of America; 2 Department of Behavioral Science, Hokkaido University, Sapporo, Japan; University of Sheffield, United Kingdom

## Abstract

The pay-it-forward reciprocity is a type of cooperative behavior that people who have benefited from others return favors to third parties other than the benefactors, thus pushing forward a cascade of kindness. The phenomenon of the pay-it-forward reciprocity is ubiquitous, yet how it evolves to be part of human sociality has not been fully understood. We develop an evolutionary dynamics model to investigate how network homophily influences the evolution of the pay-it-forward reciprocity. Manipulating the extent to which actors carrying the same behavioral trait are linked in networks, the computer simulation model shows that strong network homophily helps consolidate the adaptive advantage of cooperation, yet introducing some heterophily to the formation of network helps advance cooperation's scale further. Our model enriches the literature of inclusive fitness theory by demonstrating the conditions under which cooperation or reciprocity can be selected for in evolution when social interaction is not confined exclusively to relatives.

## Introduction

Reciprocity has long been recognized as an important constituent of human sociality that functions to lubricate social and economic exchange [1]–[2]. Reciprocity takes many forms depending on the relationship between the beneficiary and the benefactor. When a person does another a favor and receives a benefit in return directly from the same person, it is called direct reciprocity—in this case, reciprocal relationship is dyadic [3]. When the sender and the recipient of favors involve more than two parties, reciprocity goes beyond a dyad and involves a group or a network. This is referred to as indirect reciprocity [4] or generalized exchange [5]. In this case, person A delivers a benefit to person B and receives a benefit from a different person C. Different theories attempt account for why indirect reciprocity can be sustained. One line of arguments attributes C's kindness made to A to C's gratitude toward a favor he received earlier from a different third party. Indirect reciprocity operating in this manner can be portrayed as a process whereby one helps another, who in turn helps yet a different person, forming a pay-it-forward cascade [6]–[8]. In the biological literature, this kind of indirect reciprocity is also termed upstream reciprocity [9] or generalized reciprocity [10]–[11].

Our daily life is full of examples of the pay-it-forward reciprocity. For example, people hold the door for those coming afterwards in public places [12]. One reason to why the norm is held is that people walking ahead of us hold the door for us first, and in return we reciprocate by acting the same to people following us. In charity donation, a real story shows that family members of a father who benefits from organ donation of a stranger are eager to sign up as prospective organ donors, in the hope that they can be of someone else's help in the future [13]. Experimental studies convey a similar message that subjects are more willing to help if they have been helped before [14]. Finally, as a fictional yet quite illuminating example, the movie “Pay it Forward” portraits how kindness as a campaign can initiate from a naïve child to the whole community [15].

Given the ubiquity of the pay-it-forward phenomenon, an important question to be addressed is how it evolves to be part of human sociality. Explaining the evolution of reciprocity is challenging as a reciprocator does not seem to fare better than an opportunist who receive favors without reciprocating. The difference in welfare would make opportunism more advantageous, thus eliminating the survival of reciprocity. It leads us to wonder when reciprocity would triumph over self-interest. Investigating the circumstances under which indirect reciprocity evolves has become a core research endeavor in evolutionary biology [4], [16]–[17].

In stark contrast to direct reciprocity where interaction is limited to a dyad, the pay-it-forward reciprocity is conducted in a web of social interactions, which typically can be represented by network. In the network, a tie designates the flow of benefit from one node to the other. Cooperation unfolds in networks when a cooperator initiates providing favors to his network neighbors and the recipients of favor reciprocate by acting similarly to their network neighbors, pushing forward the reciprocity cascade. The problem is that reciprocity could not continue as long as there is any defector in the chain of the pay-it-forward process. Hence, how cooperators and defectors are spatially distributed in networks is critical to how far reciprocity cascading spreads.

Boyd and Richerson [18] is arguably the first study to discuss the pay-it-forward reciprocity. Their model considers a ring structure and shows that reciprocity (termed the upstream tit-for-tat in their paper) is possible to emerge only when group size is small. Nowak and Roch [9] conceptually distinguish two forms of indirect reciprocity and derive the mathematical condition for the pay-it-forward behavior (called upstream reciprocity in their paper) to evolve. A major finding of their mathematical model shows that upstream reciprocity provides a beneficial condition for cooperation to evolve if coupled with direct reciprocity. In [9], [18], a special ring structure is under investigation. A recent simulation study [19] extends the model to different networks beyond a ring.

The above models were proposed with different focuses: [18] is concerned about group size; [9] investigates the effect of coupling two forms of reciprocity, and [19] examines the effect of network topology. It is noteworthy that although the abovementioned models all address network effects to different extents, none of them is concerned with the issue of how actors of different kinds are linked in networks. Given a network, actors can be placed on the network in different manners, and the spatial arrangement could render unexpected advantages or disadvantages to the survival of reciprocity in evolutionary dynamics. A recent study that investigates the effect of interactive assortment shows that generalized reciprocity (or the pay-it-forward reciprocity termed here) is possible to evolve in groups when reciprocators interact with the like more often than random chance [10]. In this paper, we continue a similar research endeavor to study how network homophily—the degree to which actors are linked with the like in networks [20]—influences the evolution of the pay-it-forward reciprocity. Our model differs from [10] by considering a larger strategy space and local behavioral adaptation in networks. We derive an interesting finding that strong network homophily helps consolidate the adaptive advantage of cooperation, but some heterophily helps promote cooperation to a higher scale.

## Results

### The Model

Nowak and Roch proposed a “helping game” to model the pay-it-forward problem [9] (see below). We draw on this helping game to construct an evolutionary dynamics model comprising three components: a population of actors, social networks, and a rule that governs actors' behavioral adaptation.

#### Population

Two behavioral traits dissect the population into four groups: the first trait (*p*) governs the behavior of initiating helping, and the second trait (*q*) controls behavior of reciprocity. With *p* = 1, actors deliver help, and do nothing with *p* = 0. When *q* = 1, actors reciprocate and do nothing with *q* = 0. Four types of actors or four different strategies are generated accordingly: perfect cooperators (PC) for p = 1, q = 1, classical cooperators (CC) for p = 1, q = 0, reciprocal cooperators (RC) for p = 0, q = 1, and defectors (D) for p = 0, q = 0.

#### Adaptation of Behavior

Intermittently along the course of evolutionary dynamics, actors update their behavior in reference to their network neighbors. They search for the actor in their network neighborhood (including themselves) with the highest accumulated payoff and imitate his behavioral traits. If more than one actor possesses the highest payoff, one of them is randomly selected for imitation. This “learning-from-the-best” adaptation rule is also termed ‘imitation updating’ [21]. For robustness testing, we also consider other adaptation rules reported in the following section.

#### Social Network

Actors are placed on a network with ties linked to a subset of the population. The structure of links determines the potential recipients of help. When updating behavior, actors use the same network to look for targets for imitation. In this paper, we consider a particular network structure—the regular square lattice—as the baseline network. The regular square lattice with periodic boundary condition (a torus) has been intensively studied in biology. Research on the so-called “spatial game theory” has showed that lattice structure helps preserve cooperation in clusters against the invasion of defection [22]–[24]. In a regular lattice, each node has the same number of ties (nodal degree). Even though ties are possible to be rewired, described as follows, so that the prefect uniformity in nodal degree cannot maintain, difference in nodal degree is kept small over the network. This helps us to tease out the effect of heterogeneity in nodal degree on the evolution of reciprocity as was noted in [19].

#### Network Homophily

**Figure 1:**
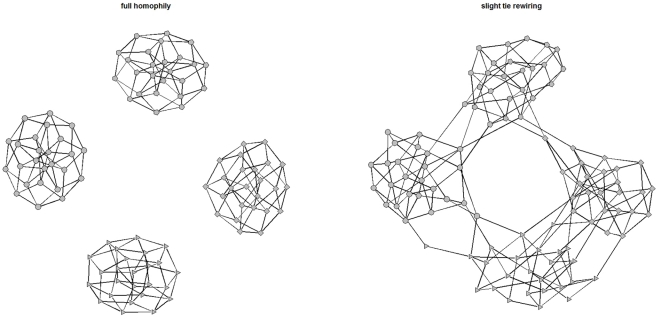
Networks before and after ties are rewired. **Left panel:** Four isolated tori, each characterized by a nodal shape. **Right panel:** Slight tie-rewiring to neighboring groups generates some inter-group ties.

We set up a torus network component of equal size for each group defined in Table 1. As a baseline, the four tori are isolated from one another, representing full network homophily, where actors of the same type are linked exclusively to one another (illustrated in Figure 1 left panel). New networks are generated to loosen up network homophily by rewiring some of the intra-group ties to be inter-group ties (see Figure 1 right panel). Through tie rewiring, we change the ecology of a node's network neighborhood without changing network density [25]–[26], thereby eliminating the effect of network density on the evolution of reciprocity. Tie rewiring is governed by a vector of probabilities that sums up to 1. To be more specific, consider the four groups defined in Table 1. For each torus component specific to group 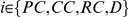, each tie of the component is checked, and with probability 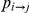, the tie that at present connects a pair of nodes in torus component *i* is reconnected to a member in torus *j*. It follows that with probability 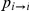 the tie stays intact, and 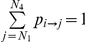, where 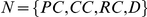. Note that each group has its own set of probabilities governing the direction of tie rewiring, independent of how ties are rewired in other groups.

**Table 1:** The Four Types of Actors in the Helping Game.

		Return help received?	
		Yes	No
Initiate providing help?	Yes	Perfect Cooperator	Classical Cooperator
	No	Reciprocal Cooperator	Defector

**Figure 2:**
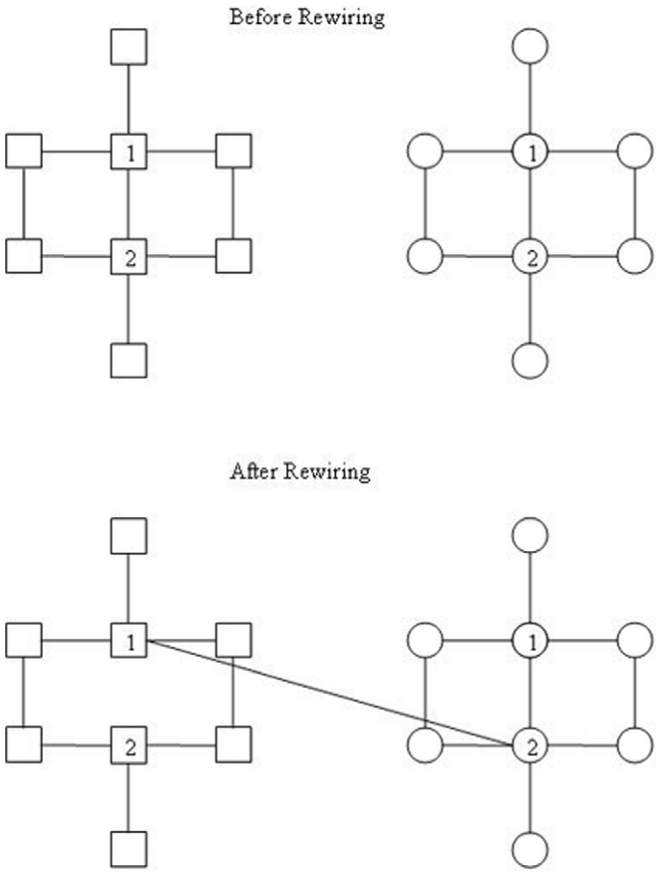
Illustration of the rule of rewiring ties. **Upper panel:** Square shaped nodes represent one group (strategy) while circle shaped nodes represent another. All actors are indexed consecutively in their original torus components. The figure shows the joint network neighborhoods of actor 1 and 2; nodes and edges beyond the joint neighborhoods are omitted. **Lower panel:** If the tie of square 1-square 2 is prompted to rewire to the circle group, one of the end nodes, here node 2, is detached from the tie, and the other node, node 1, is reconnected to circle node 2.

We index every node in each baseline torus. When a tie is prompted to rewire, it detaches one end of the tie and reconnects the other to a node in a different group with the same index number as the detached node (see Figure 2 for illustration). Which node is detached and which is reconnected is a random choice. In so doing, we minimize the impact of tie rewiring on causing large difference in nodal degree.

#### The Helping Game

We model the evolutionary dynamics of the helping game embedded in networks. In each round, actors first decide whether to deliver help. Actors with *p* = 1 incur a cost *c* and randomly pick **one** network neighbor as the beneficiary of help worth *b*. Actors with *q* = 1 reciprocate the favors, if any, received from network neighbors. They compute how many times they were helped in the previous round and repay the same amount of help to a random set of network neighbors (sampling with replacement). Initiating and reciprocating favors are synchronous in each round so actors reciprocate favors received in the **previous** round. At the end of every *s* round, each actor searches for actor in his network neighborhood (including oneself) who has the highest accumulated payoff. He inherits this most “successful” actor's behavior traits. The simulation is terminated when no occurrence of behavioral imitation persists continuously for *y* rounds or repetitive cycles are found. Once the simulation stops, we record the share of each type of actor (or strategy) in the population.

### Evolutionary Dynamics of Two Groups

Before we discuss the results of the four groups defined in Table 1, it is beneficial to gain some preliminary insights through studying the evolution of two groups (strategies) pair-wisely across the four groups. We simulate a population of two groups with equal share (1/2). Each group adopts a unique strategy. The probability of tie rewiring in one group is *p_1_* and *p_2_* in the other. Manipulating *p_1_* and *p_2_* from 0 to 1, we examine the share of each strategy at the end of the evolution.

#### Perfect Cooperator vs. Defector

**Figure 3:**
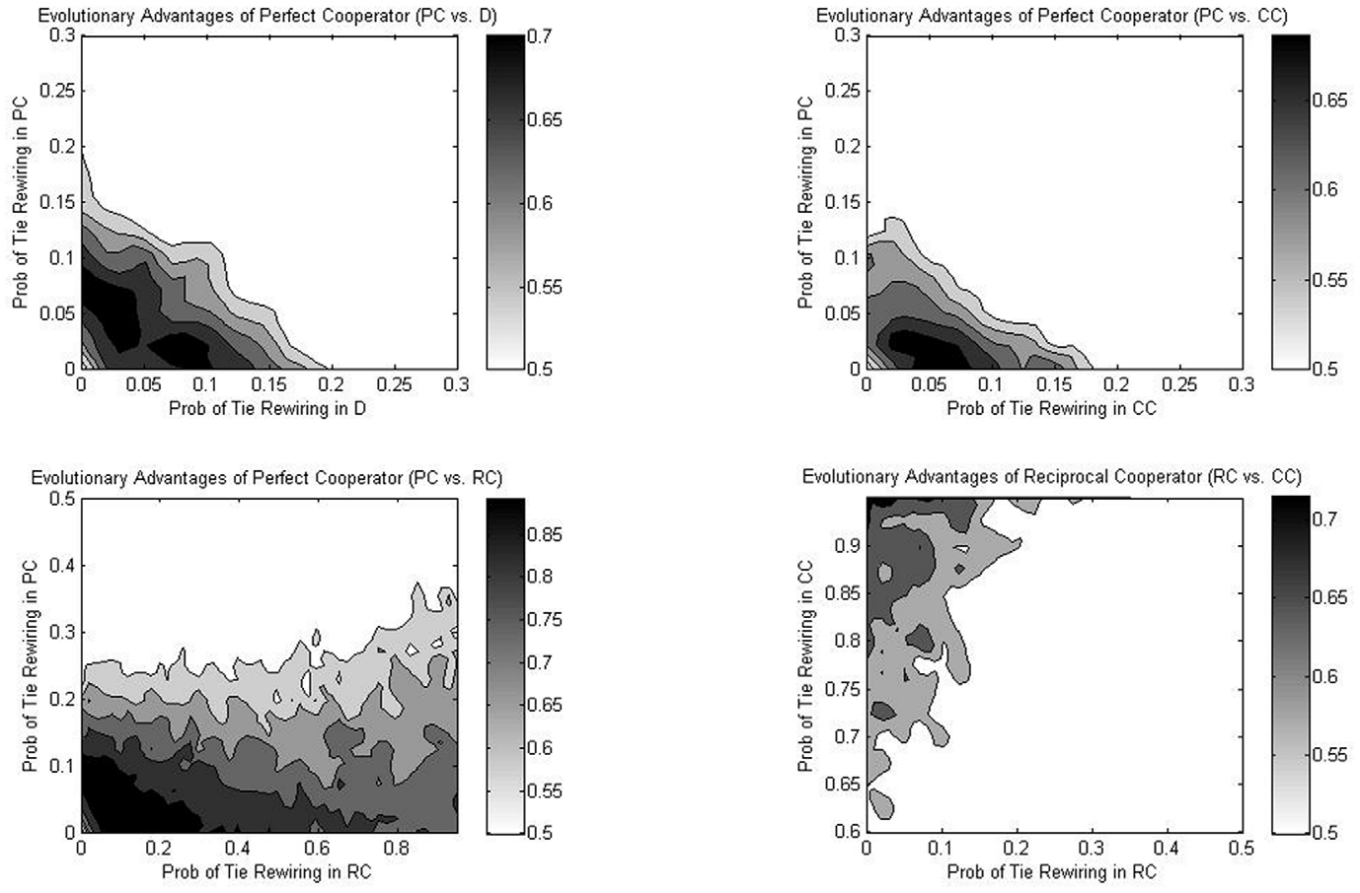
The evolutionary advantages of different kinds of actors. **Upper left panel:** The evolutionary advantages of **perfect** cooperators over **defectors**. The contour plot quantifies the average share of perfect cooperators over 100 cases. Share below 0.5 is in white color, representing inferiority when competing with defectors. The two axes mark rewiring probabilities with an increment of 0.025. **Upper right panel:** The evolutionary advantages of **perfect** cooperators over **classical** cooperators. Share below 0.5 is in white color, representing inferiority when competing with classical cooperators. **Lower left panel:** The evolutionary advantages of **perfect** cooperators over **reciprocal** cooperators. Share below 0.5 is in white color, representing inferiority when competing with reciprocal cooperators. **Lower right panel:** The evolutionary advantages of **reciprocal** cooperators over **classical** cooperators. Share below 0.5 is in white color, representing inferiority when competing with classical cooperators.

Defectors dominate prefect cooperators in most networks investigated. It makes sense because defectors receive benefits without reciprocating while perfect cooperators pay the cost of providing favors to others. Evolutionary theory shows that cooperators can survive when they interact assortatively with one another [27] or form clusters in networks [22]. Our simulation result confirms the finding: as is shown in Figure 3 (upper-left panel), perfect cooperators end up with a share higher than one-half when they are cohesive to one another, i.e., they do not experience too much tie rewiring and instead keep most of their ties linked to themselves. A few “bridging” ties to other groups help cooperators to increase popularity. Increasing inter-group ties is beneficial only when the degree of network homophily is high—after some critical point, more inter-group ties can no longer benefit the selection of cooperators in evolution.

#### Perfect Cooperator vs. Classical Cooperator

The competition between perfect cooperators and classical cooperators is similar to that between perfect cooperator and defectors. Like defectors, classical cooperators never reciprocate so perfect cooperators are disadvantaged when interacting with classical cooperators. In Figure 3 (upper right panel), perfect cooperators dominate the population when both groups do not experience much tie rewiring, leaving only a few inter-group ties linked in between.

#### Perfect Cooperator vs. Reciprocal Cooperator

Perfect cooperators have relatively higher popularity when interacting with reciprocal cooperators. This is due to the fact that reciprocal cooperators respond to prefect cooperators' favors, and therefore perfect cooperators are not as disadvantaged as when they face the other two types of actors. Similarly, perfect cooperators dominate when they are cohesive, but different from the two cases above, it does not require that reciprocal cooperators be cohesive as well.

#### Classical Cooperator vs. Reciprocal Cooperator

Figure 3 (lower right panel) shows that classical cooperators dominate reciprocal cooperators most of the time. Because classical cooperators initiate helping and reciprocal cooperators respond, the two groups seem to be equally advantageous. Scrutiny of the simulation result shows that indeed **on average** the two groups perform equally well, but the variation in payoff is larger among classical cooperators than among reciprocal cooperators. It implies that actors with the highest fitness level are more likely to be classical cooperators. Since the current model uses a learning-from-the-local-best adaptation rule, classical cooperators are more likely to be the target for imitation. Reciprocal cooperators, on the other hand, dominate when they are cohesive while classical cooperators are not.

#### Other Relationships

Defectors completely dominate classical cooperators as the latter benefit the former without receiving anything in return. Unlike perfect cooperators, classical cooperators do not return favors to one another, failing to accumulate sufficient payoffs through reciprocation to outperform the advantages of defectors derived from exploitation on cooperators. No action takes place in a world comprising defectors and reciprocal cooperators only as in this case no one initiates helping and triggers reciprocation.

### Evolutionary Dynamics of Four Groups

We now discuss the model of four groups. Recall we introduce a set of probabilities, amounting to 1, to govern the direction of tie rewiring for each group (structured as a torus component) and thereby generate new networks with different degrees of network homophily. Our goal is to investigate what probability set results in high share of each group in evolution. Exhausting the union of four sets of probabilities, one for each group, is in essence implausible. For example, suppose we divide the probability space from 0 to 1 in 10 intervals (0, 0.1, 0.2,…,1). For each group (strategy) *i*, under the constraint that 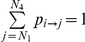, there are 275 possible probability sets. Then for four groups, the total number of possible probability sets would be 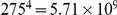. In light of the challenge, we use genetic algorithm to save the computational burden [28].

The operation of genetic algorithm can be briefly described as follows. Let matrix *P*, a 4 by 4 matrix, collects the four sets of probabilities that govern tie rewiring. Each row represents the probability set for a group. The algorithm starts with a sample pool of *P*. Without loss of generality, we consider the following probability sets as the initial condition: {0.25, 0.25, 0.25, 0.25}, {0.5, 0.5, 0, 0}, {0, 0, 0.5, 0.5} and {0, 0.5, 0.5, 0}, which represent, respectively, uniform, right-skewed, left-skewed, and central-peaked distributions. Each group can be assigned one of the four probability sets so the total number of matrix *P* considered for the first generation is 4^4^ = 256. Each matrix *P* in the pool generates a network, and we run the evolutionary model of the helping game on the network. We then check how well a network performs with respect to the popularity of the kind of strategy investigated. In reference to the performance record, the next generation of *P* would be produced over the following steps: (1) Randomly select two *P* matrices from the pool (with replacement) with probability in proportion to their performances (2) For the two selected matrices, denoted *P_i_* and *P_j_*, randomly choose two rows from one matrix to pair up with the alternative two rows chosen from the other matrix. A new matrix *P* is thus formed by inheriting half of *P_i_* and half of *P_j_* (3). Allow mutation to occur to the new matrix by randomly selecting one row and one element to uplift it by 0.1. Choose a different element of the same row to reduce it by 0.1. Mutation follows the constraint that that probability of rewiring be not over 1 or below 0.

**Figure 4:**
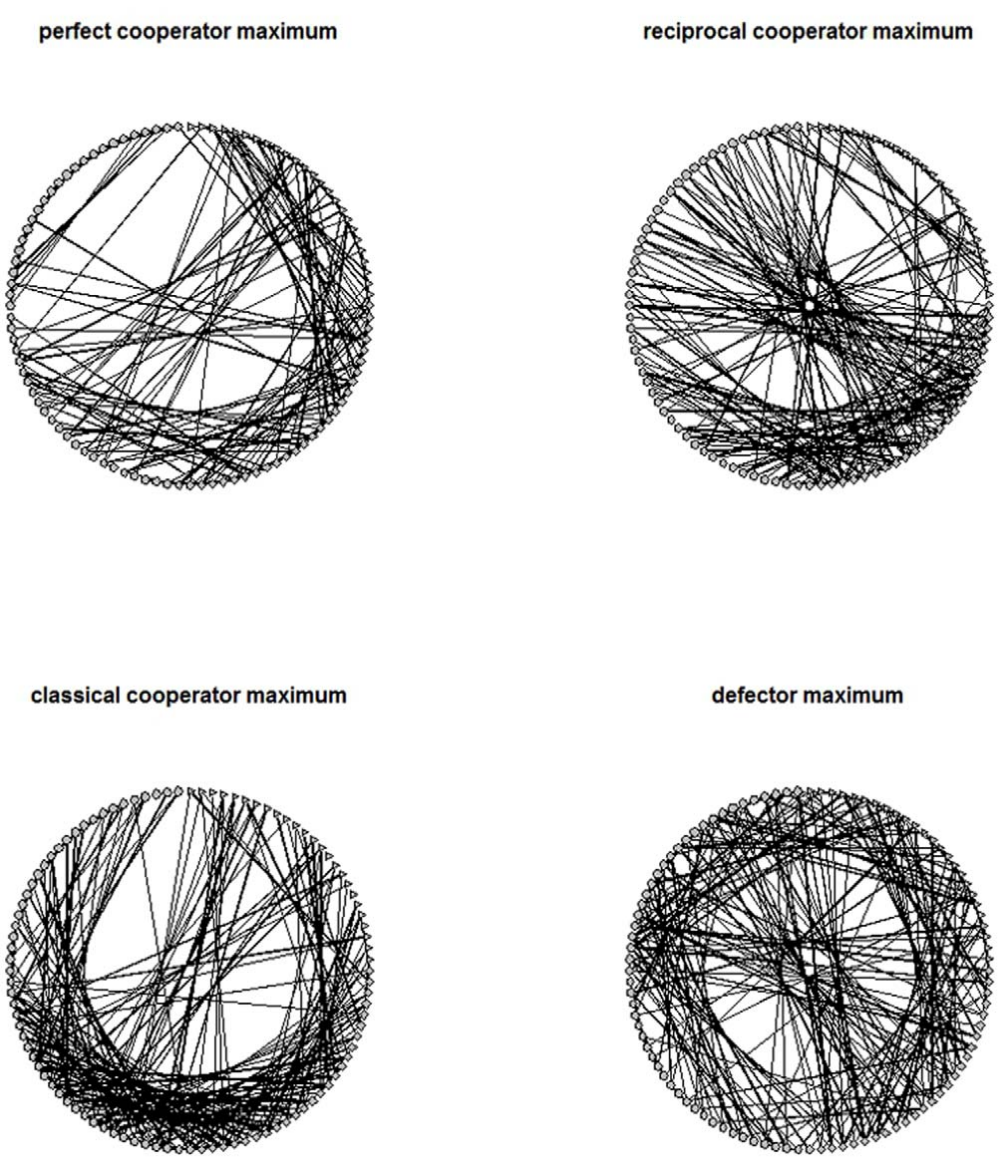
Graphic illustration of the optimal networks. **Upper left panel:** A representative network generated by following the optimal rewiring strategy selected by genetic algorithm to maximize the popularity of **prefect** cooperators. Clockwise from the tiptop of the layout: the first quadrant are nodes of defectors, followed by the second quadrant of reciprocal cooperators, the third quadrant of classical cooperators and finally the fourth quadrant of perfect cooperators. **Upper right panel:** A representative network generated by following the optimal rewiring strategy selected by genetic algorithm to maximize the popularity of **reciprocal** cooperators. The layout of nodes is same as above. **Lower left panel:** A representative network generated by following the optimal rewiring strategy selected by genetic algorithm to maximize the popularity of **classical** cooperators. The layout of nodes is same as above. **Lower right panel:** A representative network generated by following the optimal rewiring strategy selected by genetic algorithm to maximize the popularity of **defectors**. The layout of nodes is same as above.

When genetic algorithm is run, average performance of the pool of *P* would improve as generations accumulate. In each generation, we target the *P* that performs the best with respect to the popularity of each strategy. We run the genetic algorithm for 100 generations, before which the share of each strategy is found to converge to a fixed level. For each strategy, we select the best ten matrices *P* and report their means and standard deviations in Table 2. For better illustration of the data, representative networks generated by following these rewiring principles are shown in Figure 4.

**Table 2:** Means and standard deviations (in parentheses) of the ten optimal matrices *P* for each type of actor.

	Matrices P that achieve the maximum share of:			
	D(average share = 0.99)	RC(average share = 0.78)	CC(average share = 0.93)	PC(average share = 0.70)
*P* ***_D→_***_*D*_	0.06 (0.08)	0.69 (0.08)	0.11 (0.06)	0.25 (0.10)
*P* ***_D→_***_*RC*_	0.37 (0.10)	0.14 (0.07)	0.11 (0.08)	0.51 (0.19)
*P* ***_D→_***_*CC*_	0.31 (0.10)	0.10 (0.05)	0.74 (0.06)	0.24 (0.16)
*P* ***_D→_***_*PC*_	0.26 (0.13)	0.07 (0.04)	0.04 (0.02)	0 (0)
*P* ***_RC→_***_*D*_	0.68 (0.11)	0.02 (0.04)	0.01 (0.03)	0.06 (0.07)
*P* ***_RC→_***_*RC*_	0.30 (0.11)	0.73 (0.08)	0.11 (0.06)	0.83 (0.08)
*P* ***_RC→_***_*CC*_	0 (0)	0.10 (0.05)	0.84 (0.07)	0.11 (0.11)
*P* ***_RC→_***_*PC*_	0.02 (0.04)	0.15 (0.11)	0.04 (0.05)	0 (0)
*P* ***_CC→_***_*D*_	0.66 (0.11)	0.18 (0.04)	0.04 (0.05)	0.09 (0.06)
*P* ***_CC→_***_*RC*_	0.11 (0.14)	0.76 (0.07)	0.12 (0.09)	0.70 (0.08)
*P* ***_CC→_***_*CC*_	0.10 (0.07)	0.06 (0.07)	0.68 (0.06)	0.21 (0.07)
*P* ***_CC→_***_*PC*_	0.13 (0.10)	0 (0)	0.16 (0.07)	0 (0)
*P* ***_PC→_***_*D*_	0.62 (0.19)	0.00 (0)	0.00 (0)	0 (0)
*P* ***_PC→_***_*RC*_	0.07 (0.05)	0.83 (0.08)	0.08 (0.09)	0.10 (0)
*P* ***_PC→_***_*CC*_	0.06 (0.06)	0.00 (0)	0.74 (0.11)	0 (0)
*P* ***_PC→_***_*PC*_	0.25 (0.21)	0.17 (0.08)	0.18 (0.06)	0.90 (0)

The simulation results suggest that the population of perfect cooperators can grow as high as roughly 70% in networks that have the following properties: (1) perfect cooperators are cohesive to one another, leaving only a few ties linked to reciprocal cooperators; (2) reciprocal cooperators are cohesive, and (3) classical cooperators are highly linked with reciprocal cooperators. The first fact is consistent with the two-group case studied earlier: reciprocal cooperators are perfect cooperators' best partner as perfect cooperators are more advantaged when facing reciprocal cooperators than the other two types of actors. The second and the third facts, consistent with earlier findings as well (Figure 3), ensure that reciprocal cooperators be sustained in early stages of the evolution. This is important as survival of reciprocal cooperators is important to the expansion of perfect cooperators as they are a bridging group to convert the other two types of actors into perfect cooperators. The simulation data (not reported) shows that reciprocal cooperators are the first group in time to be converted to perfect cooperators, followed by classical cooperators and defectors. The proportion of conversion of each group follows the same order.

The share of reciprocal cooperators can be as high as 78% if (1) they are cohesive to one another; and (2) perfect and classical cooperators, but not defectors, are strongly attached to them. Both classical and perfect cooperators are help initiators. Upon receiving the benefits from the two types of actors, if most of reciprocal cooperators' ties are directed to themselves, they reciprocate by doing favors to one another, which invokes further rounds of reciprocity, and the virtuous circle provides an advantage in adaptive fitness to reciprocal cooperators [29], [10]. Classical cooperators can take a proportion as high as 93% if all actors are highly linked to them. A similar condition applies to the prosperity of defectors with one exception— to gain popularity, it is required that defectors not link to one another. This makes sense as the encounter of two defectors does not benefit the adaptive value of each other.

In sum, a selection of optimal tie-rewiring probability sets screened by genetic algorithm over a hundred generations shows that the prosperity of the four types of actors each requires somewhat different conditions. For classical cooperators, the key lies in attraction of ties from all types of actors, including themselves. The same principle applies to reciprocal cooperators, except that defectors must be shunned in this case. Similarly, defectors prosper by having a dense network with all other types of actors, but not themselves. In contrast, perfect cooperators need to be isolated in structure, and their expansion is channeled through a few bridging ties with reciprocal cooperators. Group cohesiveness, i.e., ties with same group members, is required for prosperity of each type of actor except for defectors. Only classical cooperators can prosper with a dense network with defectors. Finally, it is noteworthy that prosperity of one type of actor is determined in part by how other types of actors are structured—manifesting the property of interdependency in complex systems.

### Notes on Robustness of the Results and Limitations of the Model

The simulation results reported in Table 2 attempt to illustrate the general conditions beneficial to the popularity of each type of strategy. Using genetic algorithm, we attempt to locate the probability sets, wrapped up in matrix *P*, that would maximize the share of each strategy in the population. As a typical optimization problem, we cannot be sure that the optimal matrix *P* is unique, nor is there any perfect algorithm that guarantees all the optimal solutions be located. In light of these facts, one should treat the numeric information reported in Table 2 as a general trend rather than a unique optimal condition. Nevertheless, the consistency in results between the four-group and the two-group cases, wherein full probability space is considered, confirms the merit of using genetic algorithm for search of optimal solutions.

The simulation results presented here are built on a set of parameters whose values are subject to modifications. We run more simulations reported below to see how the results change when some of the modeling assumptions are relaxed, parameter values are adjusted, or alternative solutions are considered.

#### Regarding b (benefit of help) and c (cost of helping)

It is well established in mathematical/evolutionary biology that the benefit-cost ratio (*b*/*c*) of helping is critical to the selection of cooperation. A simple rule, derived originally from Hamilton [30], predicts that cooperators are more likely to survive if the benefit-cost ratio is large, other conditions being equal. We manipulate the magnitude of *b*, leaving other conditions fixed in the simulation model, to test how the benefit-cost ratio influences the results. For each type of strategy (Table 1), we adopt the corresponding optimal network-tie-rewiring probability matrix (Table 2) as the network generation principle. We then replicate five-hundred random cases of the pay-it-forward game dynamics. The online supporting material (Figure S1, Figure S2, Figure S3 and Figure S4) reports the average share of each strategy over different values of *b*. Clearly, increasing *b* leads to higher proportions of perfect and reciprocal cooperators in the population. The effect, however, is not as pronounced on the growth of defectors and classical cooperators.

#### Regarding network density

In the current model, we consider a regular square lattice network with the Neumann neighborhood (a torus) as the default network (before tie rewiring takes place). In the Neumann neighborhood, each actor has four network neighbors. When a torus is projected on a (two-dimensional) plane, each actor is neighboring others who have one-unit of distance moving on the two coordinates. We can increase network density by allowing actors to link to others further away on the plane. Widening the neighborhood radius from one (Neumann neighborhood) to four increases the number of neighbors per actor from 4 to 24 (full network in the current model setting with N = 100). We run more simulations, changing network density in this manner, to see how network density influences the results. The online supporting material (Figure S1, Figure S2, Figure S3 and Figure S4) shows that increasing network density does not impede, but instead benefits the selection of perfect and reciprocal cooperators. The finding is opposite to the general rule discussed in network-reciprocity modeling [4]: cooperation thrives if *b/c>k*, where k represents average number of network neighbors per actor. The discrepancy in the effect of network density is due to the difference in modeling assumption: in modeling cooperation game on graphs (or network reciprocity defined in [4]), an actor interacts with **each** of his network neighbors, while in the pay-it-forward game studied in earlier work [9], [19] and here, a helper randomly picks **one** network neighbor as the recipient of help. It is easy to see that increasing network density inflicts heavier burden on cooperators in the former case as a cooperator needs incur the cost of helping for each neighbor. While costly in the network reciprocity game, higher network density renders more opportunities to receive favors from others in the pay-it-forward game studied here. Reciprocating these favors, albeit costly, form a virtuous circle by reinforcing the evolutionary advantages of cooperators when they are structured cohesively in networks. It explains why there is a difference in the effect of network density between the two models of the evolution of cooperation.

#### Regarding the adaptation rule

Throughout the paper thus far, we use a deterministic learning-from-the-local-best adaptation rule. Its empirical validity recently has received some support from behavioral experiments [31].Certainly this is not the only adaptation rule available for consideration. To see whether adaptation rules make a difference in results, we run more simulations, each of which adopts a unique adaptation process currently studied in the literature, leaving other conditions same as specified in the method section. We refer to a recent study [32] that summarizes a great variety of combinations of adaptation rules and update dynamics. Three adaptation rules considered here are: Best Imitation—a deterministic adaptation rule that imitates the strategy of the most successful neighbor in fitness (the default model of the paper); Fermi function—a stochastic adaptation rule that transforms exponentially the difference in fitness to the probability of strategy imitation, and Linear Probability Payoff Difference—a stochastic adaptation rule that transforms linearly the difference in fitness to the probability of strategy imitation. Moreover, two update dynamics are investigated: synchronous updating—in each round, all actors first play the pay-it-forward game, followed by strategy adaptation, and asynchronous updating—actors take turns playing the game and updating strategies. The online supporting material (Table S1, Table S2, Table S3 and Table S4) shows how each strategy performs in different combinations of adaptation rules and update dynamics. The tables report average share of each strategy in networks generated by following their optimal tie-rewiring probability sets respectively reported in Table 2. In general, asynchronous updating is more beneficial than synchronous updating to the increase of each strategy. Among the adaptation rules considered here, Fermi function works the best, followed by best imitation and linear probability function. Thus, the result presented earlier in the paper, considering a deterministic best imitation rule, represents a modest estimation of the evolutionary result.

There could be a difference, however, between learning-from-the-*local*-best and learning-from-the-*global*-best adaptation rule. Actors under the former rule learn from the most successful one in their local network neighborhoods, while under the latter rule, they target the most successful one in the whole population. Learning from the local best is a less radical selection rule in evolutionary dynamics, and it helps preserve cooperation in locality. As we can imagine, if actors possess local vision only, a cluster of cooperators would learn from each other and remain cooperators despite the fact that a few defectors on the skirt of the cluster might fare better by exploiting cooperators positioned on the periphery of the cluster. However, under certain circumstances local adaptation is not beneficial to the selection of cooperation. It has been shown that when adaptation is made locally, altruism not only benefits a rival's fitness, but also increases his evolutionary advantages in taking over the neighborhoods which they both compete for [33]–[35]. To modify Hamilton's rule to this case, we need to consider the extra cost of altruism associated with increasing the competiveness of neighboring rivals [35]. Without loss of generality, the current paper assumes that actors have local vision both in playing the cooperation game and behavioral adaptation. It is rather rare to see actors have a local vision in one thing, yet a global one in the other.

#### Other issues

Varying population size is not expected to change the results as long as the share of each strategy stays fixed in the initial condition. Note that in the current model we start with four isolated tori—one for each strategy—before ties are rewired. Each strategy, instead of clustering in one torus, could begin with a bunch of tori of smaller sizes. For this inquiry, we run three separate simulations with the same N = 576. In one simulation, each strategy starts structured in 16 tori of size 9; in another simulation, each strategy is in 4 tori of size 36, and in the last simulation, each strategy is in 1 torus of size 144. We run 500 cases for each condition. The results show a weak effect of group size: the share of a strategy increases slightly when it starts with a smaller number of tori of larger sizes. For example, for perfect cooperators, the share is 0.59 in the first condition, 0.66 in the second and 0.68 in the third.

Other technical details that control the simulation process, such as the interval of behavioral updating (*s*) and the continuum of no imitation occurring (*y*) used to stop the simulation, do not make differences to the results.

## Discussion

Anecdotes about daily-life activities and research findings from laboratory experiments have confirmed the universality of the pay-it-forward reciprocity in human nature. What is less clear is how this kind of prosocial behavior comes into being given its inferiority in adaptive value than self-interested acts. More than two decades ago, Boyd and Richerson [18] drew a pessimistic conclusion based on their evolutionary model that the pay-it-forward reciprocity is not possible to emerge except in small groups. Yet, a recent study [10] shows that generalized reciprocity is possible to emerge so long as assortative interaction is implemented. In this article, we argue that the pay-it-forward reciprocity could prevail in large populations structured by networks under certain spatial arrangements. Following the framework of [9], we develop an evolutionary model and examine how network homophily influences the evolution of the pay-it-forward reciprocity. The simulation results carry two main messages: first, actors who inherit the behavioral trait of reciprocity need to be cohesive to one another in order to resist the invasion of defectors or non-reciprocal cooperators. However, contacts with non-reciprocator groups, albeit slightly, is important to further increasing the popularity of reciprocity. The first principle helps consolidate the adaptive advantage of cooperators, while the second principle helps market cooperation to defectors or non-reciprocators. These two principles taken together imply that moderate heterogeneity in the demographic composition of network neighborhood is beneficial to the emergence of cooperation. This insight is consistent with modeling work on cooperation along a ring structure [36], collective action dynamics [37], the evolution of social norms [38], and empirical finding of the spread of contraceptive use in social networks [39].

The first principle that cohesion is necessary for reciprocators to be adaptive is in line with the previous research on inclusive fitness that stems from the original work on kin selection [31]. According to the theory of kin selection, altruistic behavior is adaptive when *rb*-*c*>0, where *c* is the cost, *b* is the benefit, and *r* is the relatedness of the two actors. This simple inequality means that higher levels of cooperation are attained when *r* or *b* is higher while *c* is lower. This simple rule has been shown to explain altruistic behavior in a wide range of social conditions [40]. Two possible mechanisms through which a high *r* could arise between individuals are kin discrimination and limited dispersal [41]. Kin discrimination means that an individual can distinguish between relatives and non-relatives and preferentially direct cooperation towards relatives. Limited dispersal means that offspring are born and live around parents [30]. As an unintended consequence, relatedness becomes higher among interacting individuals. It means that even if individuals engage in altruistic behavior indiscriminately, their targets are likely to be their relatives. Our model echoes the limited dispersal argument of inclusive fitness theory although the inclusive fitness costs and benefits of cooperation in viscous population can cancel out, unless the scales of interaction and selection are different [33]–[35]. This is because the more likely cooperators interact with one another, despite higher benefits being achieved, the less likely defectors outside the clusters of cooperators can be reached and adapted to cooperation. Our model shows that adequate interaction with a different kind of actors does not impede, but instead benefits the increase of cooperation. It is not only because contacts with heterogeneous others help propagate the adaptive advantages of cooperation to non-cooperators, but also because the evolutionary advantages of cooperation stem in part from cohesive interactions across two different, yet mutually beneficial types of actors, such as perfect cooperators and reciprocal cooperators in the pay-it-forward game. Our model thus enriches inclusive fitness theory by demonstrating some conditions under which interaction with non-relatives can be beneficial for cooperators to be adaptive.

Network homophily—the tendency of agents to form social relationships with those with similar attributes or backgrounds—is a universal phenomenon in various domains, ranging from the formation of friendship in high schools [42] to partner choice in marriage [43]. It is unclear why humans possess a strong propensity to associate with the like, but in the study on group cooperation and coordination, research shows that homophily helps reduce communication cost and signify group identity, thereby facilitating cooperation within groups [44]. The benefit of increasing group welfare can explain in part why homophily becomes a strong psychological principle guiding the formation of social relationships. However, cooperation induced by group homophily is limited to same-group members. Larger-scale cooperation, such as combating global warming, would inevitably require alliances that go beyond local group boundaries. As is pointed out in this article, the ingredient of moderate heterogeneity added to a homophilous environment sometimes does not hamper, but instead benefits the propagation of cooperation, although how heterogeneous is seen as optimal depends on the social context in question.

The current study hopes to stimulate more empirical research on the network foundation for the emergence of the pay-it-forward reciprocity. As is emphasized in this article, not only is the typology of networks, but also the spatial distribution of heterogeneous actors is critical to how far the conduct of reciprocity spreads. To empirically test the idea, one can, for example, categorize subjects based on their prosociality propensity [45] and then manipulate the spatial distribution of these subjects in network settings conducted either in the laboratory or in the field. Feedback from the empirical research and new models modified according to it will enhance our understanding of how human morality emerges.

## Materials and Methods

### List of Parameter Values (for the simulation results reported in the context)

Population size (*N*) = 100

Benefit of help (*b*) = 5

Cost of helping (*c*) = 3

Simulation is stopped if no imitation occurs for (*y*) = 200 rounds

Behaviors are consider for adaptation for every (*s*) = 5 rounds

Number of replications for each rewiring probability set: 50

The pseudo-code of the simulation model can be found in the online supporting material (File S1).

## Supporting Information

Figure S1 **Average share of defectors over different levels of** ***b*** **and network density.**
(DOC)

Figure S2 **Average share of reciprocal cooperators over different levels of** ***b*** **and network density.**
(DOC)

Figure S3 **Average share of classical cooperators over different levels of** ***b*** **and network density.**
(DOC)

Figure S4 **Average share of perfect cooperators over different levels of** ***b*** **and network density.**
(DOC)

Table S1 **Average share of defectors in different combinations of update dynamics and adaptation rules.**
(DOC)

Table S2 **Average share of reciprocal cooperators in different combinations of update dynamics and adaptation rules.**
(DOC)

Table S3 **Average share of classical cooperators in different combinations of update dynamics and adaptation rules.**
(DOC)

Table S4 **Average share of perfect cooperators in different combinations of update dynamics and adaptation rules.**
(DOC)
