# Supplementary material for: Network Homophily and the Evolution of the Pay-It-Forward Reciprocity
Source: PLoS One. 2011 Dec 15;6(12):e29188. doi: 10.1371/journal.pone.0029188 (PMC3240652; doi:10.1371/journal.pone.0029188)
Supplement: Table S1 — Average share of defectors in different combinations of update dynamics and adaptation rules. (DOC) [file pone.0029188.s005.doc]

| Update Dynamics  Adaptation Rule | *Synchronous* | *Asynchronous* |
| --- | --- | --- |
| *Best Imitation* | 0.99 | 0.99 |
| *Fermi Function* | 0.99 | 1 |
| *Linear Probability of Payoff Difference* | 0.98 | 1 |
