# Supplementary material for: Network Homophily and the Evolution of the Pay-It-Forward Reciprocity
Source: PLoS One. 2011 Dec 15;6(12):e29188. doi: 10.1371/journal.pone.0029188 (PMC3240652; doi:10.1371/journal.pone.0029188)
Supplement: Supplementary file 7 [file pone.0029188.s003.doc]

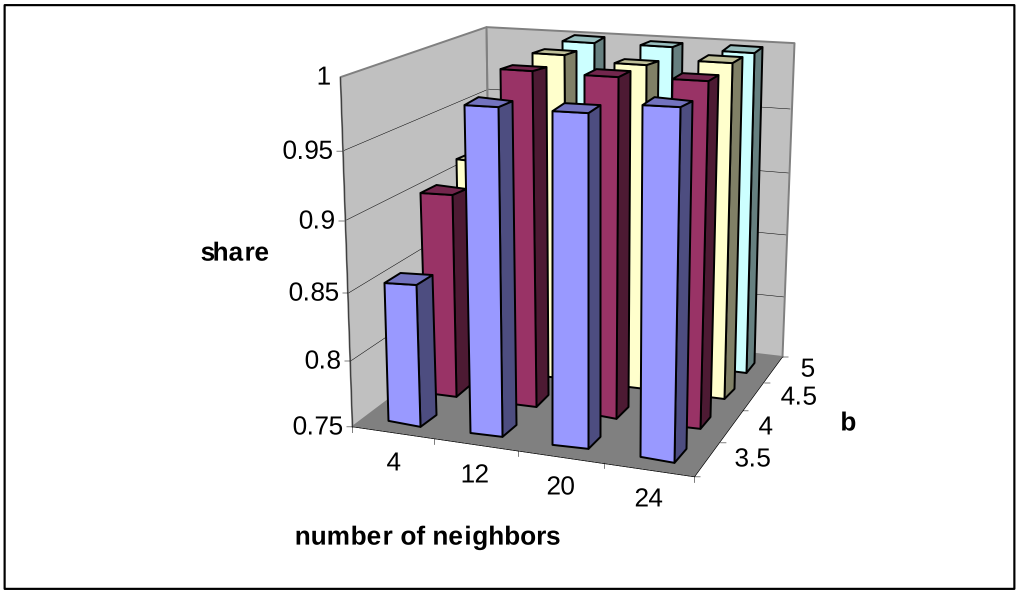
share
1
0.95
0.9
0.85
0.8
0.75
4
12
20
24
number of neighbors
5
4.5
4
3.5
b
