# Supplementary material for: Network Homophily and the Evolution of the Pay-It-Forward Reciprocity
Source: PLoS One. 2011 Dec 15;6(12):e29188. doi: 10.1371/journal.pone.0029188 (PMC3240652; doi:10.1371/journal.pone.0029188)
Supplement: Supplementary file 8 [file pone.0029188.s004.doc]

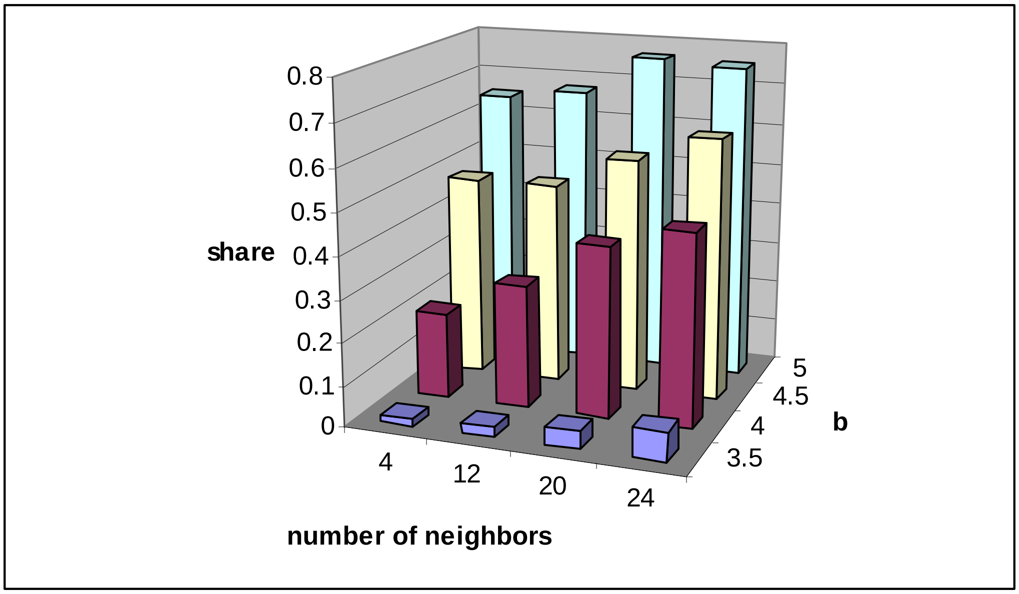
share
0.8
0.7
0.6
0.5
0.4
0.3
0.2
0.1
0
4
12
20
24
number of neighbors
5
4.5
4
3.5
b
